# Cloning of the Bisucaberin B Biosynthetic Gene Cluster from the Marine Bacterium *Tenacibaculum mesophilum*, and Heterologous Production of Bisucaberin B

**DOI:** 10.3390/md16090342

**Published:** 2018-09-19

**Authors:** Masaki J. Fujita, Yusuke Goto, Ryuichi Sakai

**Affiliations:** Graduate School of Fisheries Sciences, Hokkaido University, Hakodate 041-8611, Japan; yusuke.gotoh.1124@gmail.com (Y.G.); ryu.sakai@fish.hokudai.ac.jp (R.S.)

**Keywords:** siderophore, biosynthesis, heterologous production, bisucaberin, desferrioxamine

## Abstract

The biosynthetic gene cluster for bisucaberin B (**1**, *bsb* gene cluster), an *N*-hydroxy-*N*-succinyl diamine (HSD)-based siderophore, was cloned from the marine bacterium *Tenacibaculum mesophilum*, originated from a marine sponge. The *bsb* gene cluster consists of six open reading frames (ORFs), in contrast to the four ORFs typically seen in biosynthetic gene clusters of the related molecules. Heterologous expression of the key enzyme, BsbD2, which is responsible for the final biosynthetic step of **1** resulted in production of bisucaberin B (**1**), but not bisucaberin (**2**) a macrocyclic counterpart of **1**. To date, numbers of related enzymes producing macrocyclic analogues have been reported, but this work represents the first example of the HSD-based siderophore biosynthetic enzyme which exclusively produces a linear molecule rather than macrocyclic counterparts.

## 1. Introduction

Siderophores are microbial products that strongly chelate ferric ions, facilitating the acquisition of iron in iron-deficient environments. Iron is essential for the growth of almost all organisms and represents a key limiting factor in bioproduction; thus microorganisms utilize a variety of siderophores to compete for iron [1]. It is also reported that some bacteria utilize exogenous siderophores for their own growth, a process that has been termed “siderophore piracy” [2,3,4]. Recent work has revealed that avaroferrin (**3**), an *N*-hydroxy-*N*-succinyl diamine (HSD)-based siderophore that is produced by a marine bacterium, halts swarming of a competitive bacterium [5]. These observations suggest that siderophores contribute to complex chemical communications among environmental microorganisms.

**Figure 1:**
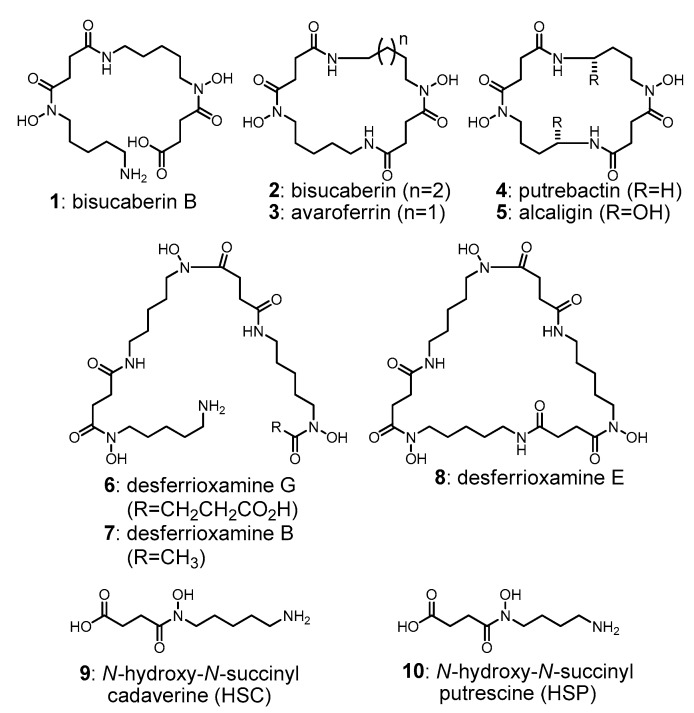
Structures of the representative *N*-hydroxy-*N*-succinyl diamine (HSD)-based siderophores (**1**–**8**) produced by various bacterial phyla, and their monomeric precursors *N*-hydroxy-*N*-succinyl cadaverine and *N*-hydroxy-*N*-succinyl putrescin (**9** and **10**, respectively). Compounds **2**–**5** and **8** are cyclic dimers and trimers, while **1** and **6** are linear dimer and trimer, respectively. Compound **7** was pseudo trimer, one of its terminals was capped by acetate group.

Desferrioxamines and related molecules (**1**–**8**) are representative bacterial siderophores composed primarily of *N*-hydroxy-*N*-succinyl cadaverine (HSC, **9**) and *N*-hydroxy-*N*-succinyl putrescine (HSP, **10**) subunits, and have been isolated from various bacterial phyla (Figure 1) [6,7,8,9,10]. Desferrioxamine B (**7**) is used clinically as a treatment for iron poisoning [11] and several interesting bioactivities are reported for this group of molecules, including inhibition of mycobacterium biofilm formation [12] as well as enhancement of macrophage-mediated cancer cell cytolysis [13].

**Figure 2:**
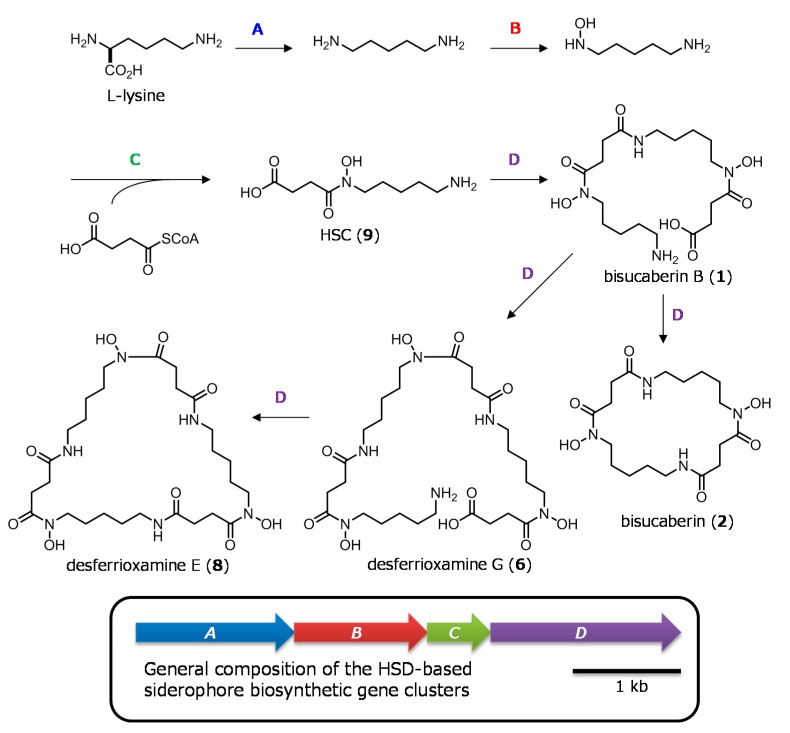
Proposed biosynthetic pathway of *N*-hydroxy-*N*-succinyl diamine (HSD)-based siderophores, and schematic organization of the typical biosynthetic gene cluster; this organization is conserved in a wide range of bacterial phyla.

To date, several gene clusters responsible for the biosynthesis of HSD-based siderophores have been cloned [14,15,16,17,18,19]. These clusters generally encode four proteins, with the first three enzymes (enzymes A–C) catalyzing the formation of the common key intermediate, HSDs (**9** and **10**), from amino acids (lysine and ornithine) by sequential decarboxylation, *N*-hydroxylation, and condensation with succinyl-CoA. The fourth enzyme (enzyme D) catalyzes the formation of multiple amide-bond linkage between the HSD monomers (**9**, **10**) as well as the subsequent head-to-tail cyclization reaction, yielding the final macrocyclic products (Figure 2) [20]. These amide bond-formation enzymes (enzyme Ds) comprise a novel group of non-ribosomal peptide synthetases [20]. It is also reported that enzyme Ds accept a wide range of HSDs as their substrates to produce a variety of macrocyclic final products with diverse bioactivities [21,22,23,24], but for which the molecular mechanisms of the regulation of the oligomerization reactions and macrocyclization reaction are largely unknown.

During the survey of novel siderophores from marine bacteria, we found a marine bacterium *Tenacibaculum mesophilum*, which belongs to the phylum Bacteroidetes isolated from an unidentified Palauan marine sponge, that exclusively produces a linear HSD-based siderophore, bisucaberin B (**1**), but does not produce the macrocyclic counterpart **2** [10]. Other bacteria reported so far produce mainly the macrocyclic forms; linear molecules are thought to be biosynthetic intermediates or shunt by-products (Figure 2). These observations suggested that *T. mesophilum* may encode the first example of a HSD-based siderophore biosynthetic machinery that inherently lacks the final macrocyclizing activity. Therefore, we expected that a detailed analysis of the enzymes from *T. mesophilum* would provide information regarding the molecular mechanism of the macrocyclization reaction. Here, we report cloning of the biosynthetic gene cluster of bisucaberin B (**1**) from *T. mesophilum*, confirmation of the enzymatic function by heterologous expression, and a brief sequential analysis of the cloned enzymes.

## 2. Results and Discussion

### 2.1. Cloning of the Bisucaberin B Biosynthetic Gene Cluster

**Figure 3:**
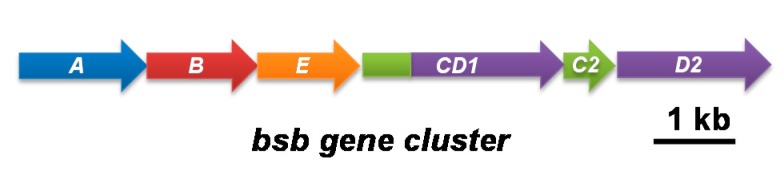
Composition of the biosynthetic gene cluster of bisucaberin B (*bsb* cluster) from a marine bacterium *T. mesophilum*, belonging to the phylum Bacteroidetes.

Though HSD-based siderophore biosynthetic genes have not been reported from phylum Bacteroidetes, taking advantage of the highly conserved amino acid sequence among amide-bond formation enzymes (enzyme Ds) from diverse bacterial taxa, we cloned the conserved part by PCR amplification using a degenerate primer set. An amplified DNA fragment from the genomic DNA of *T. mesophilum* showed high similarity to the known biosynthetic genes, suggesting the presence of a similar gene cluster in this species. We therefore screened a genomic library of *T. mesophilum* (consisting of approximately 9.6 × 10^4^ fosmid clones) using the PCR amplification screening method [25] to identify a clone containing the whole gene cluster. Shotgun sequencing of a hit clone revealed the presence of a gene cluster (total length, 8840 bp) capable of encoding a putative bisucaberin B (**1**) biosynthetic enzymes, and designated as *bsb* (bisucaberin B) cluster (Figure 3 and Table 1; accession number LC090204). The cluster contained six open reading frames (ORFs; *bsbA*, *B*, *CD1*, *C2*, *D2*, and *E*) instead of the four genes (*A* to *D*) typically observed in other related biosynthetic gene clusters reported to date. 

The first two genes, *bsbA* and *bsbB*, encode enzymes having high sequence homology to 2,4-diaminobutylate decarboxylase and l-lysine-6-monooxygenase, respectively. These two enzymes are responsible for the production of a common intermediate, *N*-hydroxy-1,5-diaminopentane, which is formed by decarboxylation of lysine followed by *N*-hydroxylation (Figure 2). The third gene, *bsbE*, encodes a protein annotated as a major facilitator superfamily (MFS) protein, that is a member of a large group of membrane transporter proteins [26]. This type of gene has not been found in other known HSD-based siderophore biosynthetic gene clusters. One of the main functions of the MFS family is transportation/exportation of small molecules such as antibiotics, thereby contributing to drug resistance. We therefore hypothesize that BsbE likely facilitates the secretion of compound **1** from the cell [27,28,29].

The most notable difference between *bsb* and related clusters was the redundancy of the DNA sequences corresponding to genes *C* and *D*. In the *bsb* cluster, there are two sets of orthologues showing homology to acyl transferases (enzyme C) and amide-bond formation enzymes (enzyme D), as shown in Table 1. Notably, one set of orthologues forms a single chimeric ORF encoding a putative bifunctional enzyme (BsbCD1) that consists of distinct N-terminal (204 amino acid residues) and C-terminal (606 amino acid residues) domains that exhibit homology to acyl transferases (BsbC1 part, indicated in green) and amide-bond formation enzymes (BsbD1 part, indicated in purple), respectively (Figure 3 and Table 1). Sequence identity between BsbC1 part and BsbC2 was 44%, and both enzymes showed 32% identity with MbsC, a corresponding enzyme encoded by a bisucaberin (**2**) biosynthetic gene cluster from a marine metagenome [18]. Amino acid identity between putative amide-bond formation enzymes BsbD1 part and BsbD2 was 50%, and those enzymes showed 42 and 46% identities to MbsD, respectively (Table 1). Thus, both the BsbCD1 and BsbC2–D2 sets are candidate enzymes for the biosynthesis of compound **1**. However, only one set of enzymes C and D would be needed for production of **1**, considering the structure and biosynthesis scheme of **1** (Figure 1 and Figure 2). Furthermore, it is reported that single enzyme Ds are capable of catalyzing multiple amide-bonds formation in the biosynthesis of other related macrocyclic molecules (Figure 2) [20]. Therefore, redundancy in the genes *C* and *D* in this cluster is of particular interest.

### 2.2. Heterologous Production of Bisucaberin B by a Fusion Gene Cluster System

**Figure 4:**
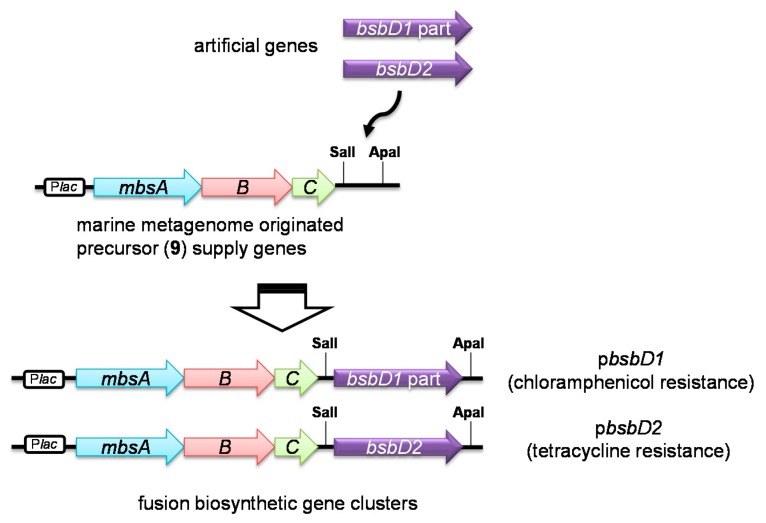
Construction of the fusion gene clusters consisting of *mbsA–C* and *bsbD1* part or *bsbD2*.

To gain further insight into the ‘redundant’ biosynthetic gene cluster, we assessed the function of putative amide-bond formation enzymes, BsbD1 part and BsbD2, by heterologous expression using a fusion gene cluster system that we have developed previously (Figure 4) [19,30]. Three genes (*mbsA–C*), originally cloned from a marine metagenome as bisucaberin (**2**) biosynthetic genes, were combined in this system with a SalI-ApaI cloning site that permits gene *D* insertion by cassette method. Encoded enzymes (MbsA–C) provide the key precursor HSC (**9**); thus insertion of gene *D* would proceed further reactions to produce HSD-based siderophores depending on the catalytic property of the inserted gene *D*s (Figure 2). Artificial genes of *bsbD1* part and *bsbD2* whose sequences were optimized for *E. coli* expression (see Supplementary Materials) were synthesized and individually ligated into the fusion gene cluster system to form p*bsbD1* (mbsA–C + bsbD1 part) and p*bsbD2* (mbsA–C + bsbD2), respectively (Figure 4). These constructs were separately or simultaneously transformed into competent *E. coli* to prepare a total three clones, two of which were single transformants bearing either p*bsbD1* part or p*bsbD2*, and one of which was a double transformant containing both plasmids.

**Figure 5:**
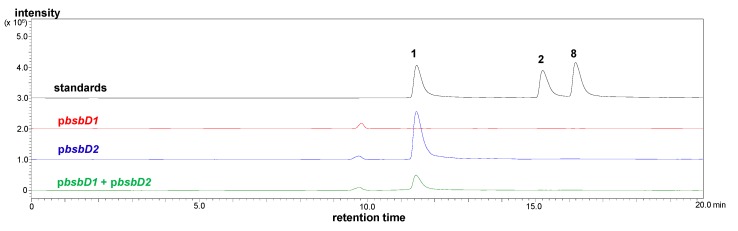
Ion current chromatograms of the culture broth of each clone and a mixture of standards (compounds **1**, **2**, **8**).

The siderophore production was monitored by Chrome Azurol S (CAS) test [31] as well as high performance liquid chromatography (HPLC) with mass spectrometer (MS) detector. In the CAS assay, siderophore activity was detected from the culture broths of the p*bsbD2* clone and double transformant, but not from that of the p*bsbD1* clone. LC-MS analyses confirmed the presence of bisucaberin B (**1**) only in the CAS-active culture broths (Figure 5), and activity guided isolation and NMR analysis unambiguously identified that CAS active product was bisucaberin B (**1**, Supplementary Materials Figures S1 and S2, Table S1). Production of compound **1** by the p*bsbD2* clone was quantified by LC-MS to be 16.1 mg/L, which was comparable to that of the original producer, *T. mesophilum* (38.9 mg/L) [10]. Double transformation decreased production of **1** to about 30% of the p*bsbD2* single transformant (Figure 5), probably due to the competitive expression of active BsbD2 and inactive BsbD1 part. Neither macrocyclic dimer bisucaberin (**2**) nor trimer desferrioxamine E (**8**) were detected from the culture broth of any transformants (Figure 5). Other candidate linear siderophores, desferrioxamine G (**6**) and B (**7**) were also not found from any clones (Supplementary Materials Figure S3). The above results indicated that BsbD2 efficiently and exclusively produced compound **1** without any other factors such as BsbD1 part, indicating that BsbD2 is a single key enzyme in the amide-bond formation in the biosynthesis of **1** but lacks the macrocyclization function.

Although the present experiments failed to demonstrate the function of the BsbD1 part, the above results suggested that either the *bsbD1* part was not efficiently or functionally expressed in this system, or the encoded protein, the BsbD1 part, was inherently inactive. 

To date, several amide-bond forming macrocyclization enzymes (enzyme Ds) responsible for the production of HSD (**9**, **10**) based-siderophores have been experimentally characterized from various bacterial species (e.g., desferrioxamine E (**8**) synthetases DesD [15] and DfoC^C^ [19] from *Streptomyces coelicolor* and *Erwinia amylovora*, respectively; bisucaberin (**2**) synthetases BibC^C^ [16] and MbsD [18] from *Aliivibrio salmonicida* and marine metagenome, respectively; putrebactin (**4**) synthetase PubC [17] from *Shewanella* sp.; alcaligin (**5**) synthase AlcC [14] from *Bordetella pertussis*, Table 2). In the present study, we demonstrated that BsbD2 is the first example of an enzyme D that lacks the macrocyclization ability. Thus, analysis of the sequence of BsbD2 was expected to provide insight into the molecular basis for this largely unknown enzyme.

**Figure 6:**
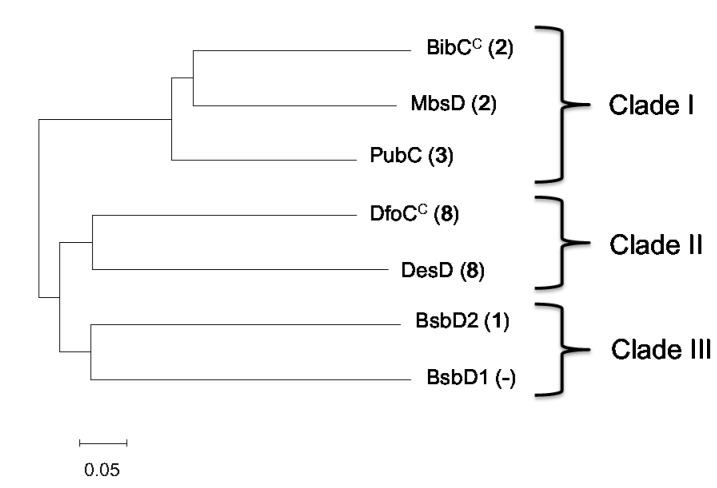
Phylogenetic analysis of the amide-bond formation enzymes by neighbor-joining method. The number inside the parentheses indicates the major final product of each enzyme.

The length of each of the known enzyme Ds (Sequences: See Supplementary Materials) is approximately 630 residues. Overall amino-acid sequence identity among the family is approximately 50% (Table 2), and BsbD2 also had similar identity/similarity to other enzymes. Phylogenetic analysis of the six enzymes (along with the BsbD1 part) revealed a correlation between the final products and the enzyme sequences (Figure 6) [32]. In the resulting phylogenetic tree, enzymes were categorized into three clades as follows. Clade I consists of macrocyclic dimer (**2** and **4**)-producing enzymes including MbsD [18], BibC^C^ [16], and PubC [17]. Clade II is a group of macrocyclic trimer (**7**)-forming enzymes, and includes DfoC^C^ [15] and DesD [19]; of note, these two enzymes originate from highly divergent bacterial taxa (gamma-Proteobacteria and Actinobacteria, respectively) suggesting that this phylogenetic tree largely reflects enzymatic function rather than bacterial taxonomy. Clade III may represent a group of exclusively linear molecule-producing enzymes, as BsbD2 falls into this clade. 

The phylogenetic tree generated from a total of 62 sequences, including 55 homologous proteins without functional characterization from bacteria belonging to all different genera, showed similar results; clade III which BsbD2 fell into was clearly separated from other clades containing macrocycle forming enzymes (Supplementary Materials Figure S4). These results suggested that linear molecule forming enzymes could be sequentially distinguishable from other macrocycle forming enzymes. However, sequential feature or characteristic amino-acid residues responsible for their discrete functions could not be identified in BsbD2 or other proteins in the same clade. Additional studies including mutation of BsbD1 and BsbD2 or heterologous expression of the homologues will reveal the relationships between sequence and function of the Clade-III enzymes.

## 3. Materials and Methods 

### 3.1. General Experimental Procedures

NMR spectra were recorded on an ECP-400 NMR spectrometer (JEOL, Tokyo, Japan) at 400 MHz for ^1^H in dimethylsulfoxide (DMSO)-*d*_6_ as a solvent. Chemical shifts of ^1^H NMR spectra were referenced to the solvent peaks: δ_H_ 2.49 for DMSO-*d*_6_. Preparative and analytical HPLC were done with a Prominence HPLC system equipped with photodiode array detector (Shimadzu, Kyoto, Japan). LC-MS analyses were done with an LCMS-8040 LC-MS system (Shimadzu). UV absorption in the CAS solution assay was measured on a SpectraMax M2 microplate reader (Molecular Devices, Sunnyvale, CA, USA). DNA sequences were determined with a BigDye terminator v3.1 cycle sequencing kit (Thermo Fisher Scientific, Waltham, MA, USA) on 3130*xl* Genetic Analyzer (Thermo Fisher Scientific). Electroporation was done with a MicroPulser electroporator (Bio-Rad, Hercules, CA, USA). A GeneAtlas thermal cycler (Astec, Fukuoka, Japan) and a KOD Plus Neo PCR kit (Toyobo, Osaka, Japan) or GoTaq Green Master Mix (Promega, Fitchburg, WI, USA) were used to amplify DNA fragments. Oligo DNAs for cloning and DNA sequencing were purchased from Hokkaido System Science (Sapporo, Japan). Plasmid vectors pBCSK+ (Agilent Technologies, Santa Clara, CA, USA), and pHY300PLK (Takara Bio, Shiga, Japan) were used for cloning. All chemicals were purchased from Wako Pure Chemical Industries (Osaka, Japan), Nacalai Tesque (Kyoto, Japan), or Takara Bio (Shiga, Japan) except for those specifically mentioned.

### 3.2. T. mesophilum Genomic Library Construction

Genomic DNA was collected from a *T. mesophilum* cell pellet (following cultivation for 4 days at 30 °C in sea water containing 5 g/L yeast extract and 10 g/L tryptone) using the DNeasy Blood & Tissue Kit (Qiagen, Hilden, Germany) according to the manufacturer’s protocol. The resulting genomic DNA was size separated by agarose gel electrophoresis (1% low melting point agarose gel, 30 V for 15 h). The DNA above 23 kb was recovered from the gel by digestion with a thermostable β-agarase (Nippon Gene, Tokyo, Japan). Purified DNA was blunt-ended using the End-It DNA End-Repair Kit (Epicentre, Madison, WI, USA), followed by ligation into pCC1FOS fosmid vector (Epicentre). This mixture was subjected to packaging with the MaxPlax Lambda Packaging Extract (Epicentre), and then transfected into Escherichia coli EPI300-T1R (Epicentre) according to the manufacturer’s protocol. Transformants were plated to Luria-Brrtani (LB) agar (10 g tryptone, 5 g yeast extract, 10 g NaCl, and 10 g agar per L of deionized water) containing chloramphenicol (30 μg/mL) to yield a *T. mesophilum* genomic library comprising a total of 9.6 × 10^4^ clones with an average insert length of 35 kb.

### 3.3. Amplification of the Fragment of the Biosynthetic Genes by Degenerate Primer PCR

An approximately 450-bp DNA fragment of the putative bisucaberin B (**1**) biosynthetic gene cluster was PCR amplified from *T. mesophilum* genomic DNA using degenerate primers. The primers (forward: 5′-GTNGCNAAYAAYGGNCGYATYGGGTT-3′, reverse: 5′-SWNARNCCRCGCATRAAACCCATRTT-3′) were designed to amplify a sequence conserved among the genes encoding the known amide-bond formation enzymes (enzymes D) of the HSD-based siderophore clusters (see Supplementary Materials Sequences S3–S9). The PCR program consisted of an initial denaturation at 95 °C for 2 min, followed by 30 cycles at 94 °C for 20 s, 50 °C for 20 s, and 72 °C for 70 s. Amplified DNA was cloned into T-vector pMD20 (Takara), and then transformed into NEB 10-beta competent cells (New England Biolabs, Ipswich, MA, USA). Cloned DNA was subjected to DNA sequencing.

### 3.4. Cloning of the Bisucaberin B Biosynthetic Gene Cluster (Bsb Cluster)

Fosmid clones containing the putative bisucaberin B (**1**) biosynthetic gene cluster were screened from a genomic library pools (consisting of ~96,000 fosmid clones) based on the PCR amplification of the above-determined sequence (primers; forward: 5′-CAGCCATCGTAAACACGC-3′, reverse: 5′-GCCTGTACATCCATGGC-3′) to yield 8 hit clones [25]. In brief, fosmid clones (~96,000) were divided into 16 pools, then whole DNA was extracted from each pool and PCR analyzed to detect the presence of the target sequence. Hit library pool which contain target sequence was divided again into smaller pools, and PCR analyzed. These steps were repeated until single clone in which bearing biosynthetic gene cluster was identified. A DNA sequence spanning 11,620 bp which contains the entire biosynthetic gene cluster was determined from one of the hit clones by shot-gun sequencing by random digestion with Sau3AI restriction enzyme then sub-cloning and DNA sequencing, followed by further sequencing by primer-walking method using dye-terminator cycle sequence kit (Thermo Fisher Scientific). Sequence analysis, including read assembly and ORF detection, was performed using Vector NTI Advance (Ver. 11; Invitrogen, Carlsbad, CA, USA). The resulting DNA sequence data was deposited in the DNA Data Bank of Japan (DDBJ) as accession number LC090240 (bisucaberin B biosynthetic gene cluster).

### 3.5. Synthesis of Artificial Genes 

Two 1833-bp sequences that were optimized for *E. coli* expression without SalI and ApaI restriction enzymes sites (see the Supplementary Materials) were designed. The first one encodes 607 amino acids of the *C*-terminal part of BsbCD1; while the second one encodes BsbD2. Designed genes were chemically synthesized (Fasmac, Kanagawa, Japan) and cloned into pUC19 via flanking SalI (upstream) and ApaI (downstream) recognition sites.

### 3.6. Construction of the Fusion Gene Clusters

The resulting artificial genes *(bsbD1* part and *bsbD2*) were excised from the vector by double digestion with SalI and ApaI, purified by agarose gel electrophoresis, and then extracted from the gel using the AxyPrep DNA Gel Extraction Kit (Axygene Biosciences, Union City, CA, USA) according to the manufacturer’s instructions. The purified *bsbD1* part and *bsbD2* DNA fragments were ligated into a modified pBCSK+ vector (Agilent Technology, Santa Clara, CA, USA) and a modified pHY300PLK vector (Takara Bio), respectively. Used vectors were modified to contain the *mbsA–C* genes, and SalI, ApaI restriction enzymes sites downstream of the *lac* promoter (Figure 5) [19,30]. The resulting constructs were designated as p*bsbD1* and p*bsbD2*.

### 3.7. Preparation of Single and Double Transformants

The p*bsbD1* and p*bsbD2* plasmids were separately transformed into NEB 10-beta *E. coli* competent cells by electroporation. Cells were plated to LB agar containing 30 μg/mL chloramphenicol or 30 μg/mL tetracycline to yield clones carrying the respective single plasmid. Electrocompetent cells were made from resulting transformants [33], then electroporated again with the complementary plasmids and plated to LB agar containing both 30 μg/mL tetracycline and 30 μg/mL chloramphenicol to yield the double transformants.

### 3.8. CAS Solution Assay

A CAS solution assay was performed according to the previously reported method [31]. Test samples were mixed with an equal volume of CAS assay solution (0.6 mM cetyltrimethylammonium bromide, 15 μM FeCl_3_, 150 mM CAS, 0.5 M piperazine, 0.75 M HCl); following incubation at room temperature for 4 h, absorption at 630 nm was measured by microplate reader.

### 3.9. Heterologous Production and Identification of Bisucaberin B

CAS active p*bsbD2* clone was pre-cultured overnight in LB medium containing tetracycline and then inoculated into four 1-L flasks, each containing 400 mL of LB medium supplemented with 30 μg/mL tetracycline and 0.1 mM IPTG. These cultures were incubated at 30 °C for 4 days with shaking at 225 rpm. After centrifugation, the CAS assay-active molecules in the supernatant were adsorbed onto C18 resin, then eluted with a stepwise aqueous-MeOH gradient system (water, 10%, 30%, 50%, 70%, and 100% MeOH). Fractions eluted with 10–30% MeOH were combined and fractionated by Sephadex LH-20 column chromatography with 50% aqueous MeOH to afford a CAS-active fraction. This fraction was further chromatographed by reversed-phase semi-preparative HPLC (Inertsil ODS-3, GL Sciences, Tokyo, Japan) with an aqueous MeOH linear gradient system from 10% to 60% over 30 min to give bisucaberin B (**1**). Identification of the product was performed by LC-MS and ^1^H NMR with comparison to authentic material (Supplementary Materials Figure S2, Table S1) [10].

### 3.10. LC-MS Analysis of Metabolites

The prepared single and double transformants were cultured at 30 °C for 4 days with shaking at 225 rpm in LB medium containing appropriate antibiotics and 0.1 mM IPTG. The resulting spent culture media were mixed with an equal volume of MeOH, then centrifuged to remove insoluble material. A portion of the supernatants were analyzed by LC-MS (column, Inertsil ODS-3, 2 mm × 100 mm (GL Sciences, Tokyo, Japan); solvents, 0% to 60% aqueous-MeOH linear gradient system with 0.2% AcOH; flow rate, 0.2 mL/min; detection: multiple reaction monitoring at *m/z* 401.2 > 201.1, 419.2 > 201.1, 563.3 > 201.1, 601.3 > 201.1, and 619.3 > 201.1).

### 3.11. Quantification of Bisucaberin B by MRM Analysis

The concentration of bisucaberin B (**1**) in the culture medium of the p*bsbD2* clone was determined by the multiple-reaction-monitoring (MRM) method using *m*/*z* 419.2 (pseudomolecular ion) as the parent ion and *m*/*z* 201.1 (HSC (**9**) ion) as the daughter ion with chromatographic conditions identical to those used above. Collision energy (−20 kV) was used for the fragmentation.

### 3.12. Phylogenetic Analysis

Phylogenetic analysis was conducted using Molecular Evolution Genetics Analysis (MEGA) version 7 software by Neighbor-Joining method [32]. Used sequences were listed in the Supplementary Materials.

## 4. Conclusions

We report here the cloning of a novel HSD-based siderophore biosynthetic gene cluster from a marine sponge-associated bacterium, *T. mesophilum*, belonging to the phylum Bacteroidetes. This cluster consists of six genes including two sets of acyl transferase (enzyme Cs) and amide-bond formation enzymes (enzyme Ds), in contrast to the four genes previously reported in this class of siderophore biosynthetic gene clusters. Using heterologous expression, we characterized that BsbD2 is the first siderophore-producing enzyme that synthesizes linear HSD-based siderophore exclusively. Phylogenetic analysis suggested that the amino acid sequence of BsbD2 could be distinguishable from other known macrocycle-producing enzymes.

## Figures and Tables

**Table 1:** Bisucaberin B (**1**) Biosynthetic Enzymes.

Proteins	Length	Annotated Functions	Identity ^3^
BsbA	502 aa	lysine decarboxylase	43%
BsbB	440 aa	lysine-6-monooxygenase	51%
BsbE	391 aa	siderophore secretion	-
BsbCD1	810 aa	acyl transferase	32% ^1^
		peptide synthetase	42% ^2^
BsbC2	196 aa	acyl transferase	32%
BsbD2	606 aa	peptide synthetase	46%

^1^ N-Terminal 204 aa; ^2^ C-Terminal 606 aa; ^3^ Sequence identity was compared with corresponding enzymes of bisucaberin (**2**) biosynthesis (MbsA–D).

**Table 2:** Sequence Identity/Similarity among the Enzyme Ds.

Proteins	BsbD2 (1)	BibC^C^ (2)	MbsD (2)	PubC (4)	DesD (8)	DfoC^C^ (8)
BsbD1 (-)	50/71	47/66	43/64	42/64	49/69	47/67
BsbD2 (**1**)	-	47/65	46/66	45/63	49/68	45/66
BibC^C^ (**2**)		-	59/75	63/76	50/69	48/65
MbsD (**2**)			-	61/75	49/69	44/63
PubC (**4**)				-	47/68	45/64
DesD (**8**)					-	56/72

Parentheses: major products; values are percentage.

## Supplementary Materials

The following are available online at http://www.mdpi.com/1660-3397/16/9/342/s1, Figure S1: ^1^H NMR spectrum of the heterologously produced bisucaberin B (**1**) in DMSO-*d_6_*, Figure S2: ^1^H NMR spectrum of the authentic bisucaberin B (**1**) in DMSO-*d_6_*, Figure S3: LC-MS chromatograms of the culture broths of each clone, Figure S4: Phylogenetic tree of the amide-bond formation enzymes and 55 function unknown homologues generated by neighbor-joining method. Table S1: ^1^H NMR data for compound 1 in DMSO-*d*^6^, Sequence S1: DNA Sequence of the artificial gene of the BsbD1 part optimized for *E. coli* expression, Sequence S2: DNA Sequence of the artificial gene of the BsbD2 optimized for *E. coli* expression, Sequence S3: Amino acid sequence of BsbD1 part, Sequence S4: Amino acid sequence of BsbD2, Sequence S5: Amino acid sequence of MbsD, Sequence S6: Amino acid sequence of BibC^C^, Sequence S7: Amino acid sequence of PubC, Sequence S8: Amino acid sequence of DesD, Sequence S9: Amino acid sequence of DfoC^C^.
