# Supplementary material for: Cloning of the Bisucaberin B Biosynthetic Gene Cluster from the Marine Bacterium Tenacibaculum mesophilum, and Heterologous Production of Bisucaberin B
Source: Mar Drugs. 2018 Sep 19;16(9):342. doi: 10.3390/md16090342 (PMC6164419; doi:10.3390/md16090342)
Supplement: Supplementary file 1 [file marinedrugs-16-00342-s001.pdf]

Supplementary Materials

Cloning of the Bisucaberin B Biosynthetic Gene Cluster from the Marine Bacterium *Tenacibaculum mesophilum*, and Heterologous Production of Bisucaberin B

Masaki J. Fujita,* Yusuke Goto, and Ryuichi Sakai

Graduate School of Fisheries Sciences, Hokkaido University, Hakodate, 041-8611, Hokkaido, Japan.

*Author to whom corresponding should be addressed;

E-mail: masakifujita@fish.hokudai.ac.jp (M.J.Fujita)
Tel: +81-138-40-8806

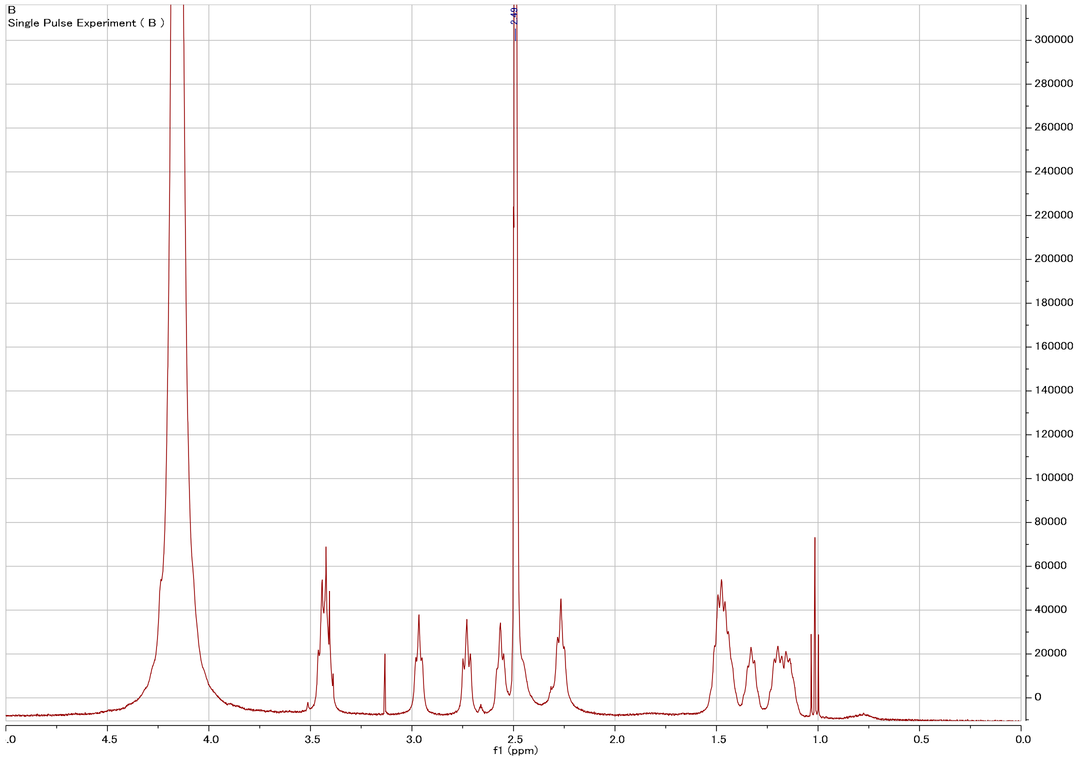

Figure S1. ^{1}H NMR spectrum of the heterologously produced bisucaberin B (**1**) in DMSO-d_6.

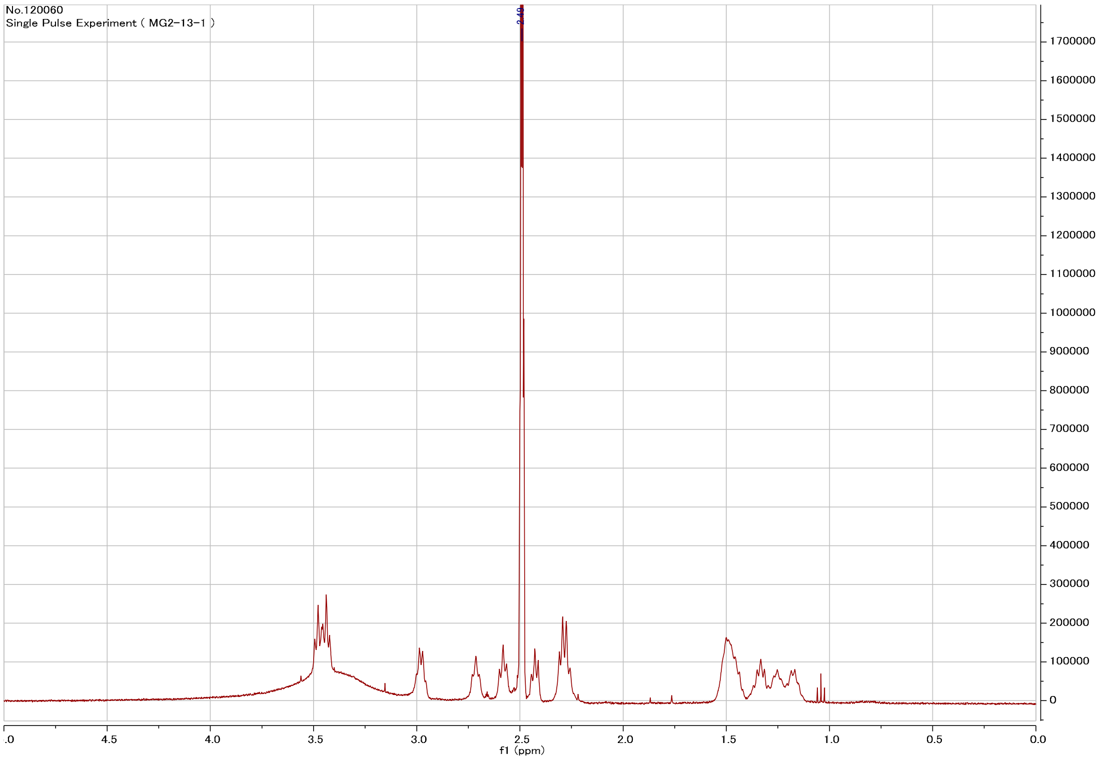

Figure S2. ^{1}H NMR spectrum of the authentic bisucaberin B (**1**) in DMSO-d_6.

Table S1. ^{1}H NMR data for compound **1** in DMSO-d_6

	δ_H mult. (J (Hz))		δ_H mult. (J (Hz))
1		1'	
2	2.44 t (6.4)	2'	2.29 t (6.4)
3	2.31 t (6.4)	3'	2.59 t (6.4)
4		4'	
5	3.45 t (6.4)	5'	3.48 t (6.4)
6	1.47[a]	6'	1.51[a]
7	1.21 quint. (7.2)	7'	1.27 quint. (6.8)
8	1.35 quint. (6.8)	8'	1.49[a]
9	2.99 quint. (6.4)	9'	2.71 t (6.4)

a: coupling was not determined due to overlapping

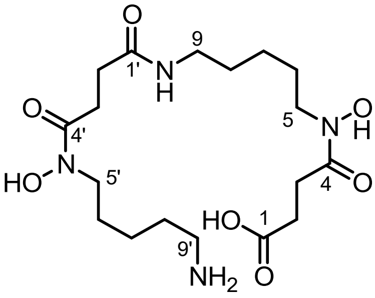

1: bisucaberin B

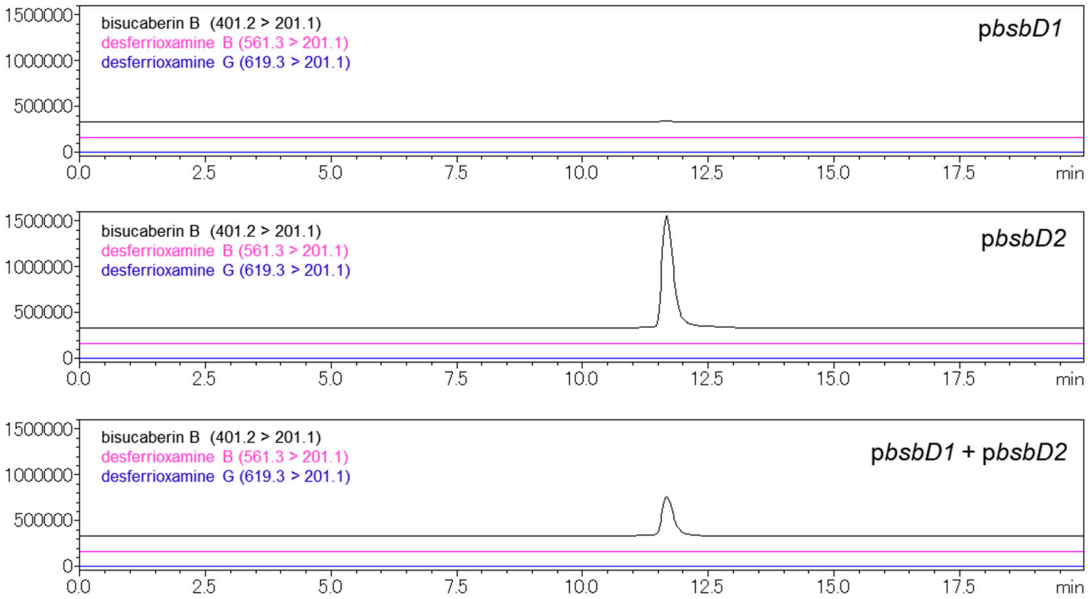

Figure S3. LC-MS chromatograms of the culture broth of each clone. Mass peaks corresponding to the linear trimers were not detected.

Sequence S1. DNA Sequence of the artificial gene of the BsbD1 part optimized for *E. coli* expression.

ATGAACACTCTGGACAACGTAGTGAGCCCTCAGCAGAGTACCCAACATATTCAGCCAAA
GGTATGGCAGAAAGCAAATCTGCTGCTGGTGAAGAAAGCGTTGTGTGAATTCTCGCATG
AACTCCTGATTCAACCCGTGATTATCCAAGAAGTGGATAATGGCTACAAACTGTATAAAG
TTTATGCCGACGATACCGAAATTCAGTATGAATTTAAAGCGAAACCGATGGCTCTGAACC
ATCTGATGATTGACGAAAATAGCATTCGCAAATACATCAAGGAAGTTGAGGAAGAAATCG
ATGCCATTACGTTTATCAAAGAATTTCGCAAAGCCCTCGGTATCGCGGATGAGAAAATGC
CGGTTTATCTCGAAGAAGTGATTTCGACGTTATATGGTAGCGCCTTTAAGATTACGAAAGG
TAATCCTACAGTGGAAGAACTCGCGACTGCGGATTTCCAGACAATTGAGCAAAGCATGA
CCGAAGGACATCCGGGCTTTGTGGCCAATAATGGTCGGATCGGCTTTGACAGCAGTGACT
ATCGCTCTTATGCGCCGGAGGCTGGTAACTCGTTTTCGTTACTGTGGCTGGCTGGCCATAA
GCTCAAAGCAGTGTTTAGTGCGATCGAGGCTCTGCCGTATGAATCACTGATCCGTCAAGA
GTTAGATGTGGACACGATCGCCCAATTCAACAAAATCATTGAGGAGAAAGGCTTTTCTCC
AAAGGACTATCTGTTTATCCCGGTTCATCCGTGGCAATGGTTTAACAAACTGGCAACCATT
TTCGCGTCAGAAGTTGCTAAGGGCGATCTGATCTGTCTCGGCTATGGACCAGATCAATATC
TGGCGCAGCAGTCGATTCGGACGCTGTTTAACATTTCCAATCCACAGAAATTTTACACCA
AAAGCGCACTCTCTATCCTGAACATGGGATTTATGCGTGGTTTACCTCTGTACTATTTGGG
GACAGCACCCAAAATGGCTGTATGGCTGGAAAATTTACTGTACAACGATGCCTATATCAA
AACCAACGGCTTTCGCATGCTGTCCGAAATTGGTTCAGTGAGTTACGTGAATCCGTACTT
CGAAGAATTCGGTCCGCACAACGATTATAACAAAATGCTGGCATCACTGTGGCGTGAAA
GCCCTTACTCCGTAGTCAAAGAGAATCAGAAACCTCTGACTATGGCCGCGTTGTTGCACA
TTGATCATTACGGCAAAGCACTGTTGCCAGAAATGATCAAAGATTCTGGGATTAGCATTG
ATAATTGGTTGCGTAGTTACCTTAAAGCGTATCTGTCTCCCATGCTTCACTGCTTCTACTAT
TACGACTTAGTCTTTATGCCACATGGGGAAAATATTATCCTTGTTCTGGAAAACAATATTC
CGGTCTATGCTCTGCTGAAAGACATTACCGAAGAAGCCTGCATTCTTAATCCGGAGGTCG
AACTTCCGGAAGAGCTTAAACGCATGTATGCGTTGGTTCCGGAGGATGTCAAACTGCTTT
CCATTTTCACCGATATGTTCGATGGCTTCTTCCGTTTCTTAGCGCCGATTCTGGAGAAACA
CGCCAACTACGGTGAACATCGCTTTTGGGAGCTGGTCGCGGAAAACATCCACGAGTACC
AAGAACAGTTCCCGGAATTGAGCGGGAAATTTAAACAGTATGACCTGTTTGCGGAAGAT
TTTAAACTGTCCTGCTTAAACCGCTTACAGCTGAACAACCACAAACAGATGATTGACCTG
GATGATCCCGTAGCCCTGCTCCAGTTCGCAGGCAAGTTGAAGAACCCGATCGCAGTTTTC
AAGAATCAGGAGGTCTAA

Sequence S2. DNA Sequence of the artificial gene of the BsbD2 optimized for *E. coli* expression.

ATGACTAACAACATTGCACACTTACAACCCAAGGTGTGGTCGTTTGTCAACCGTCAATTA

ATTAAGAAGGCCATTAGCGAATTTTCTCACGAGTTGATCCTGACGCCTGAATTCATCCTTG
AGGAAACCGATGGATGTATCTATCTGATCACCTCTGACAATAATGAGTTCACGTACCAGTT
CAAAGCGAAGAAGTACGCGTTGGACCATTGGCTTGTAGACGAGAAGAGCATTATTAAGA
AGAACAATATTAGCAACGAAGTCTACTTGGACGCGCTGCATTTCATCACAGAATTTCAGA
ATACTCTGGGCATTCCTGACGAATTTCTTGCAACGTATCTCGAAGAAATTACGAGTACCCT
GTCTGGTGCTGCTTACAAATATGTTAATGAAAAGTTTTCAGCGAATGAGCTGGCGGAAAA
GACATTTCAAGAGATCGAACATGCCATGACTGAGGGACACCCGTGTTTCGTGGCTAATAA
CGGACGTATTGGTTTCAACATTAAGGATTACCAAAAGTATGCCCCAGAATCGAACCAGTC
ATTTAAGCTCCTTTGGATCGCCGCTCATAAGAAATATGCAACTTACACCGCCGTGAAGAAT
TATGAGTATGAGAAACTTCTGGAGTCTGAACTTGGGAAAGAAAAGCTCGCATCATTCAA
AGAGGTGGTTAAGAAACAGAATGTCGCAACAGAAAACTATATCTTCATGCCAGTACACC
CCTGGCAGTGGAAGAACAAAATTGTTGCGGTCTTTGGAGCAGACATCGCACAGAAGAAT
ATCATCAGCGTAGGGGAATCTGACGATGAATTCAGCGCTCAACAATCAATTCGTACCCTC
TTTAACGCAAGCCACCCAGAAAAGCTCTATACCAAAACGGCACTGTCTATTCTGAACATG
GGTTTTATGCGCGGGCTGTCGCCATACTATATGCAATCCACCCCTCACATTACCCAGTGGA
TCACAGATCTCCTTGCCGATGATATTTACCTGCAAAACAACGGCTTCACTATGTTAGGTGA
GGTGGCAACCGCTGGATATCATAATCACTATTACGAAACTCTTGGGAAGACCAATCCGCA
CAACAAGATGTTATCTGCGCTCTGGCGCGAAAGCCCCTTCACAAAGATCAGCTCCAACC
AACGCGTATTCACGATGGCGGCATTACTGCATATTGACTATCAGGATAAGAGCCTTCTGGC
CGCGCTGATCGAAGCAAGTCCATATAATACAACTACTTGGATCCAACGTTATCTGAAGGCT
TATCTGGCGCCCTTACTTCATTGCTTCTACAAGTATGACTTTGTATTCATGCCACATGGGGA
GAACCTCATCCTTGTTCTGGAAGAGAACACCCCTGTACACGTTCTTATGAAAGACATTAC
GGAAGAGGTTATCGTTTTCAACGAGACTATGCATCTTCCGGAGCACGCTAAGCGTCTCTT
CGTCAAGACGTCTGACAAAATGAAGGTGCTGTCTATCTTCACGGATGTGTTCGACTGCTT
CTTCCGTTTCATGGCCCAACAGCTGGATTGCTATAGCTCATTCAGCGAAGATAACTTCTGG
CAACTGGTTGCCGATTGCGTCTACGAGTACCAGGAACAACACCCCGAGTTTAGCGAGAA
ATACAAGCAGTACGATCTCTTCGTTAAGGAATTTGACCGCTGCTGCCTGAATCGTCTTCA
GTTGTCAAATACGAAGCAGATGTTAAGTCTTGCCAACCCCATTGAGTCTCTTAAGTTGGA
GGGCGTGTTAAAGAATCCGCTGGCTCGTTTCAAGAAGGAAACTGTTAAAACCGTACAGT
CCCTGGAATCAATCTAA

Aino acid sequences of enzyme Ds used for phylogenetic analysis, and peptide sequences referenced for degenerate primer cloning. (Red: forward degenerate primer sites; Blue: reverse degenerate primer sites)

Sequence S3. Amino acid sequence of BsbD1 part. (accession number; LC090204)

MNTLDNVVSPQQSTQHIQPKVWQKANLLLVKKALCEFSHELLIQPVIIQEVDNGYKLYKVY

ADDTEIQYEFKAKPMALNHLMIDENSIRKYIKEVEEEIDAITFIKEFRKALGIADEKMPVYLE
EVISTLYGSAFKITKGNPTVEELATADFQTIEQSMTEGHPGFVANNGRIGFDSSDYRSYAPEAG
NSFSLLWLAGHKLKAVFSAIEALPYESLIRQELDVDTIAQFNKIIEEKGFSPKDYLFIPVHPWQ
WFNKLATIFASEVAKGDLICLGYGPDQYLAQQSIRTLFNISNPQKFYTKSALSILNMGFMRGL
PLYYLGTAPKMAVWLENLLYNDAYIKTNGFRMLSEIGSVSYVNPYFEEFGPHNDYNKMLAS
LWRESPYSVVKENQKPLTMAALLHIDHYGKALLPEMIKDSGISIDNWLRSYLKAYLSPMLH
CFYYYDLVFMPHGENIILVLENNIPVYALLKDITEEACILNPEVELPEELKRMYALVPEDVKLL
SIFTDMFDGFFRFLAPILEKHANYGEHRFWELVAENIHEYQEQFPELSGKFKQYDLFAEDFKL
SCLNRLQLNNHKQMIDLDDPVALLQFAGKLKNPIAVFKNQEV

Sequence S4. Amino acid sequence of BsbD2. (accession number; LC090204)
MTNNIAHLQPKVWSFVNRQLIKKAISEFSHELILTPEFILEETDGCIYLITSDNNEFTYQFKAK
KYALDHWLVDEKSIIKKNNISNEVYLDALHFITEFQNTLGIPDEFLATYLEEITSTLSGAAYKY
VNEKFSANELAEKTFQEIEHAMTEGHPCFVANNGRIGFNIKDYQKYAPESNQSFKLLWIAAH
KKYATYTAVKNYEYEKLLESELGKEKLASFKEVVKKQNVATENYIFMPVHPWQWKNKIVAV
FGADIAQKNIISVGESDDEFSAQQSIRTLFNASHPEKLYTKTALSILNMGFMRGLSPYYMQST
PHITQWITDLLADDIYLQNNGFTMLGEVATAGYHNHYYETLGKTNPHNKMLSALWRESPFT
KISSNQRVFTMAALLHIDYQDKSLLAALIEASPYNTTTWIQRYLKAYLAPLLHCFYKYDFVF
MPHGENLILVLEENTPVHVLMKDITEEVIVFNETMHLPEHAKRLFVKTSDKMKVLSIFTDVF
DCFFRFMAQQLDCYSSFSEDNFWQLVADCVYEYQEQHPEFSEKYKQYDLFVKEFDRCCLN
RLQLSNTKQMLSLANPIESLKLEGVLKNPLARFKKETVKTVQSLESI

Sequence S5. Amino acid sequence of MbsD**.** **(**accession number; BAL45578)
MTASIIEQTRLSHLTPAIWNQASRHLLAKILSEFSHEKLIAPELLLPAEAEQEACWQLKLDTRD
GQLCYRFSGHHYQLDHLQIAPDSIECFKDGEQQQPDAMLLIIALKERLGISDALLPTYLEEITS
TLYSKAFKLLWQAKPVQELVDCDYQQIEAAMTEGHPVFVANNGRIGFDVDDWRAFTPESG
QPLQLEWLAVSSEHTSLALIAGLDYRQLLQDELGDALLLRFEQKIRQQGKNPDDYFLMPVH
PWQWREKISRIFAADLARDRLIHLGQGRDEYQVQQSIRTFFNLSQPKRCYVKTALSILNMGF
MRGLSPYYMSRTPEINAFVAELIDSDPFFANQRFVLLREIAAIGYHHRYYEQALEKDTPYKK
MLSALWRESPYALSFQGEPLVKPGQQLMTLAALLHQDNQQQSLLAALIEASPLSAHDWMSR
LLDLYLTPLLHAFFAYDLVFMPHGENLILVLDDKVPVTILMKDIGEEVAILNGSAPLPGRVAQL
AVSLEEEMKLNYILLDIFDCIFRFMAPLLDSHTSLSEQGFWSLVAGKVRDYQASHPQFADKY
RRYDLFQPTFVRTCLNRIQLNNNQQMIDLEDREKNLRFAGDIDNPLHPFAASHSFGVTPTQA
ERLADPAL

Sequence S6. Amino acid sequence of $BibC^{C}$. (accession number; WP_012549027)

MKNSSKNPSLSLATSHLTTEYWHKANQHLIAKMITELSHEQIITPIKLDDASNAQAASWCITF
NSDTGTSEYLFRARQYQLDHLFVEPQSITCTKDDKNQPLDAVSFILSCRHLLEISDALLPTYL
EEITSTLYSKAYKLMHQNKTSAQLANASYQEIEAAMTEGHPVFIANNGRIGFDMLDHVEFSP
ESGQSLNLQWIAVLREKTSFAVIESLSYDRLIFDELGQSQLNEFNQQLSMQGLEPSHYYLMPI
HPWQWREKISRIFAADIANQYVVPLGTTEDKYQAQQSIRTFFNLSSPEKCYVKTALSILNMG
FMRGLSPYYMSRTPAINTFIANLIETDPYFAKKQFFVLKEVAAIGYHHSYYEQATRTDNPYKK
MLSSLWRESPYAPDQHGNVLVNKQQKLLTMASLLHVDDQGKSLISALMADSPLSDHNWLK
QYMDLYLQPLLHSFFAYDLVFMPHGENLILVLEDNSPIKIIMKDIGEEVAILNGEKTLPNDMN
CLAVDLEDPMKLNYILLDIFDCIFRFIAPLLEQQTQVSESDFWEIVADSVKDYQQEHPQFDAK
YQRYDLYCSSFARTCLNRIQLNNNQQMIDLEDREKNLRFAEDIANPLALFAKTHRII

Sequence S7. Amino acid sequence of PubC. (accession number; WP_011622234)
MNLATNRALLKPFTQAEFPTLEAAAHPHVPAHLMPEYWQAANRHLVKKILCEFTHEKIISPQ
IYRQAAGINHYELRLKDCTYYFSARHYQLDHLEIEAGSIRVSSAGQDKPLDAMSLIIKLKDAL
GMSETLLPTYLEEITSTLYSKAYKLAHQAIPATTLAKADYQTIEAGMTEGHPVFIANNGRIGF
DMQDYDQFAPESASALQLVWIAVRKDKTTFSSLEGLDHDSLLKQELGEQFTKFQQHLSALG
QAADSFYFMPVHPWQWREKIARTFAGEIARGDIIYLGESQDCYQVQQSIRTFFNLSAPQKCY
VKTALSILNMGFMRGLSPLYMSCTPQINAWVADLIESDSYFAEQGFVILKEIAAIGYHHRYYE
EALTQDSAYKKMLSALWRESPLPHIEPQQTLMTMAALLHVDHQEQALLAALIKHSGLSAKE
WVKRYLNLYLSPLLHAFFAYDLVFMPHGENLILVLDAGIPVKILMKDIGEEVAVLNGSEPLPQ
EVQRLAVELEEEMKLNYILLDIFDCIFRYLAPILDKQTEVSEAQFWELVADNVRDYQAQHPQ
LADKFAQYDLFKDSFVRTCLNRIQLNNNQQMIDLADREKNLRFAGGIDNPLAAFRQSHAFG
NQKLKPKS

Sequence S8. Amino acid sequence of DesD. (accession number; WP_0111028585)
MSLADAVAHLTPERWEEANRLLVRKALAEFTHERLLTPEREPDDGGGQTYVVRSDDGQTAY
RFTATVRALDHWQVDAASVTRHRDGAELPLAALDFFIELKQTLGLSDEILPVYLEEISSTLSG
TCYKLTKPQLSSAELARSGDFQAVETGMTEGHPCFVANNGRLGFGIHEYLSYAPETASPVRL
VWLAAHRSRAAFTAGVGIEYESFVRDELGAATVDRFHGVLRGRGLDPADYLLIPVHPWQW
WNKLTVTFAAEVARGHLVCLGEGDDEYLAQQSIRTFFNASHPGKHYVKTALSVLNMGFMR
GLSAAYMEATPAINDWLARLIEGDPVLKETGLSIIRERAAVGYRHLEYEQATDRYSPYRKML
AALWRESPVPSIREGETLATMASLVHQDHEGASFAGALIERSGLTPTEWLRHYLRAYYVPLL
HSFYAYDLVYMPHGENVILVLADGVVRRAVYKDIAEEIAVMDPDAVLPPEVSRIAVDVPDDK
KLLSIFTDVFDCFFRFLAANLAEEGIVTEDAFWRTVAEVTREYQESVPELADKFERYDMFAP
EFALSCLNRLQLRDNRQMVDLADPSGALQLVGTLKNPLAGR

Sequence S9. Amino acid sequence of DfoC^{C}. (accession number; GAJ89919)

MNDTHLLQAGTWLTGDNWAEANRLLIRKAIAEFAHEKIVTPAECAHGRYSLAVPGSETEYQ
FTASRLALDHWEIDAASLTKQENGHPLALDALQFITEFNEVIGIPQALLATYMEEISSTLCSSV
FKLQKNNPDSRALVNADFQTVESSMTEGHPCFVANNGRIGFDARDYLAYAPEAATPVNLIW
VAVHRRNAHFSSLSDLQYERLMREELGQSTVEQFNAQLTEKGLTHADYLFMPVHPWQWQN
KLLTVFAADIANNDIVWLGVGDDQYQAQQSIRTFFNRSHPNKRYVKTALSVLNMGFMRGLS
PYYMATTPAINEWLQDLVAGDEWLQRCDFRILREVAAVGYHNRHYEKAIKGDSAYKKMFA
ALWRDNPVAELKPGQRLMTMASFLHVDHHQKALLPALIADSGLAAERWVERYLSCYLSPLL
HCFYQHDLVFMPHGENLILLLENNVPVSAYMKDIGEEIAVMNPDAVLPEKVQRLAVDVPEN
LKLLSVFTDVFDCIFRFISAILHQSATLPEEQFWQAVARCVKEYQQAHPHLASKFSRYDMFAP
EFTRSCLNRLQLANNQQMINLSDPAENLKFAGTLVNPIARWR

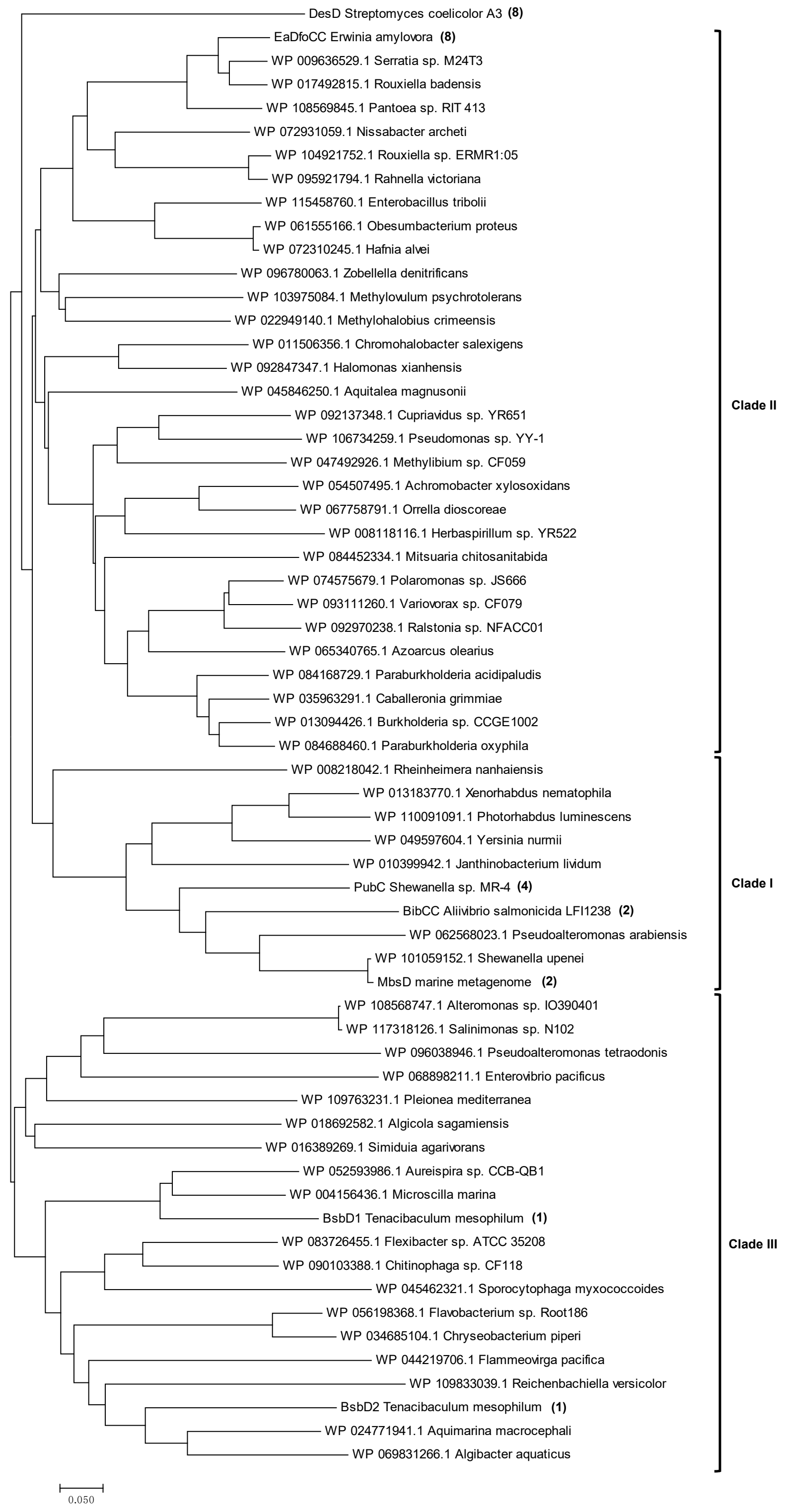

Figure S4. Phylogenetic tree of the amide-bond formation enzymes and 55 function unknown homologues generated by neighbor-joining method. The numbers inside the parentheses indicates the major final product of each enzyme.
